# Analysis of Feasibility, Adherence, and Appreciation of a Newly Developed Tele-Rehabilitation Program for People With MCI and VCI

**DOI:** 10.3389/fneur.2020.583368

**Published:** 2020-11-27

**Authors:** Irene Eleonora Mosca, Emilia Salvadori, Filippo Gerli, Laura Fabbri, Silvia Pancani, Giulia Lucidi, Gemma Lombardi, Leonardo Bocchi, Stefania Pazzi, Francesca Baglio, Federica Vannetti, Sandro Sorbi, Claudio Macchi

**Affiliations:** ^1^Istituto di Ricovero e Cura a Carattere Scientifico Fondazione Don Carlo Gnocchi, Milano, Italy; ^2^Dipartimento di Ingegneria dell'Informazione, Università degli Studi di Firenze, Firenze, Italy; ^3^Consorzio di Bioingegneria e Informatica medica–CBIM, Pavia, Italy; ^4^Dipartimento di Neuroscienze, Psicologia, Area del Farmaco e Salute del Bambino, Università degli Studi di Firenze, Firenze, Italy; ^5^Dipartimento di Medicina Sperimentale e Clinica, Università degli Studi di Firenze, Firenze, Italy

**Keywords:** mild cognitive impairment, vascular cognitive impairment, tele-rehabilitation, efficiency, web application

## Abstract

**Background:** Patients with Mild Cognitive Impairment (MCI) and Vascular Cognitive Impairment (VCI) are at a high risk of progressing to dementia. Recent guidelines indicate the importance of promoting multidimensional and multi-domain interventions to prevent further decline. Due to its growing effectiveness, comparable to conventional face-to-face interventions, the use of technology is gaining relevance. Tele-rehabilitation systems have the potential to engage patients in multi-dimensional activity programs and to guarantee a low-cost continuum of care through remote control. A possible limitation of such programs is represented by the lack of familiarization with technology and computers in elderly people. The purpose of this study is to describe the feasibility, adherence, and appreciation of the GOAL Tele-R system, administered by a web-application through remote control in patients with MCI/VCI.

**Methods:** Feasibility of the Tele-R system was evaluated by means of distribution of patients' attrition along the study phases, controlling for potential systematic bias in drop-out rates due to the technological device. Adherence was evaluated analyzing drop-out rates and indexes of carried out activities. Patients' appreciation was analyzed through *ad hoc* satisfaction questionnaire items.

**Results:** Out of 86 approached patients, 25 (29%) were not enrolled, 30 (35%) dropped-out after randomization, and 31 (36%) completed the study (standard care group *n* = 12, the tele-R group *n* = 19). Compared to the tele-R group, rates of drop-outs resulted significantly higher for the standard care group (34 vs. 62%, respectively, *p* = 0.029). Taking into account baseline characteristics, females resulted in a statistically significant higher rate of drop-outs compared to males (66 vs. 27%, respectively, *p* = 0.003). Overall adherence to the proposed activities was 84% (85% for cognitive module and 83% for physical activity module). Concerning satisfaction, participants provided a good mean level of appreciation (3.7 ± 0.8, range 1–5), a positive feedback for usability, and a subjective perception of cognitive, emotional, and physical benefits due to the training.

**Conclusion:** The GOAL Tele-R system seems a feasible technological rehabilitation program, reaching an acceptable level of adherence and appreciation in patients with an MCI/VCI condition.

**Clinical Trial Registration:** www.ClinicalTrials.gov, ID: NCT03383549 (registration date: 26/dec/2017).

## Introduction

Mild Cognitive Impairment (MCI) is a clinical syndrome that includes persons who do not fulfill a diagnosis of dementia, but who have a high risk of progressing to a dementia disorder (1). This diagnosis describes a prodromal or transitional state whereby individuals present reduced performances on selected neuropsychological tests with spared autonomies in basic and instrumental activities of daily living (1, 2). Furthermore, cerebrovascular diseases could lead to a vascular cognitive impairment (VCI), which represents a high-risk stage to develop vascular dementia (3).

No effective disease-modifying treatments are available for patients with MCI/VCI, and the most recent guidelines indicate the importance of promoting multidimensional and multi-domain interventions to prevent cognitive decline (4). A multidimensional program is characterized by the integration of different activities such as the combination of motor and cognitive activities (5). A cognition-focused intervention could involve training typically based on a set of standard tasks designed to train cognitive functions (6). The tasks may be structured according to a multidomain intervention paradigm to exercise memory, attention, language, and/or executive functions (6–8).

Evidence has shown that MCI/VCI patients who underwent cognitive training reported changes in brain activation patterns and increased connectivity between brain regions (9, 10). A meta-analysis (11) supported a significant role in MCI/VCI for physical activities to counteract cognitive decline, for all levels of physical activity. Finally, prospective studies demonstrated that socially stimulating activities could have a protective effect against dementia (12). In this regard, studies reported that the association of a cognitive, physical and social stimulation could represent a promising multidimensional intervention for patients with MCI/VCI to contrast dementia onset (13–15) and for patients with mild AD to improve cognitive-behavioral status by restoring the neural functioning (16). Unfortunately, these results can be obtained through intensive face-to-face sessions that are sometimes cost-demanding and unlikely implementable on a large scale (17). Because of its comparable effectiveness to conventional interventions, the use of technology to assist persons at risk or with early dementia is gaining relevance (17, 18). In recent years, a considerable amount of research has focused on the development of low-cost and home-based tele-rehabilitation systems to provide rehabilitation programs (19). Tele-rehabilitation may represent an effective method to gain patients' engagement and to guarantee a low-cost continuum of care through remote control (17, 20). In fact, this kind of service supplies distant support, information exchange between patients and their clinical providers and promotes the administration of multi-dimensional activity programs (20, 21). However, a possible limitation of such programs is represented by the risk of a low adherence to the treatment, due to the lack of familiarization with technology and computers in elderly people (18, 20, 22–24). In the framework of tele-rehabilitation interventions in patients with MCI, several studies have proposed the possibility to provide a tailored intervention, both in its intensity and duration (17, 18), focused on cognitive training (25, 26). Recent reviews investigated the feasibility and acceptability of telemedicine programs in older adults, finding overall positive results (27, 28). However, only a few studies were focused on the feasibility and the acceptability of a remotely delivered cognitive rehabilitation for persons with early AD (29, 30) or mild cognitive impairment (30, 31). Narasimha and Colleagues reviewed 16 usability studies among geriatric patients, suggesting overall good feasibility and usability. However, in this review, no Italian studies were included, and only one was specifically focused on patients with MCI showing several limitations (e.g., small sample size, MCI mixed with mild dementia) (28).

In Italian setting, tele-rehabilitation studies among older patients are mainly focused on healthy aging, to promote an improvement in quality of life (32–34) or on geriatric demented patients (35). Despite the existence of recommendations for the use of serious games in MCI (36, 37), to date evidence of Italian studies reporting a specific analysis of the feasibility and the adherence of tele-rehabilitation training in MCI/VCI is missing. To investigate the beneficial effects provided by both cognitive, physical and social activities, the Games for Older Adults' Active Life (“GOAL Tele-R” project) was created. Particularly, the GOAL Tele-R project aimed to propose the use of a user-friendly web application developed through a co-participatory design (38) involving patients with MCI/VCI, clinicians, technicians, and caregivers. This article is aimed at reporting data on feasibility, adherence and appreciation of the GOAL Tele-R system administered by a web-application through remote control in patients with MCI/VCI. The analysis of the effectiveness of Tele-R is not part of this work, and it is still under evaluation.

## Materials and Methods

The GOAL (Games for Older Adults' Active Life) Project is a randomized controlled clinical trial. The study methods and protocol have been previously published in detail (23). According to the inclusion criteria, eligible participants have been assessed through a baseline extensive evaluation including cognitive, behavioral, functional, and perceived quality of life measures [to see the detailed evaluation, see Fabbri et al. (23)]. Among the scales included in the baseline protocol, the following have been used in the present study:

- Montreal cognitive assessment (MoCA), as a global cognitive functioning screening tool (39);
- Free and Cued Selective Remainding Test (FCSRT);
- Digit Span forward and backward;
- Corsi Span forward and backward;
- Rey Complex Figure Test;
- Modified card sorting Test;
- Trail Making Test Part A and B;
- Stroop Test;
- Phonemic Fluency;
- Semantic Fluency.
- Activities of Daily Living Inventory (ADCS/ADL), to assess the patient's level of autonomy in the basic and instrumental activities of daily living;
- Center for Epidemiological Studies Depression scale (CESD) to evaluate depressive symptoms;
- Physical and mental component summary scores of the 36-Item Short Form Survey (SF-36) to assess the subjective perceived quality of live.

Subsequently, through the use of a computer algorithm (http://www.graphpad.com/quikcalcs/randMenu/), patients have been randomly assigned to the control or treatment group. The control group received a standard care (12 months follow-up visit). The treatment group performed, for 8 weeks, cognitive exercises 3 days a week, Adapted Physical Activities (APA, 40) 2 days a week, and social activities once a week, administered through a tablet. Immediately at the end of treatment, subjects underwent a post-training assessment including the administration of an *ad hoc* satisfaction questionnaire. The latter was developed in order to evaluate the following issues:

- experience appreciation (range: 1–5, where 5 indicated a very high appreciation);
- preference module;
- usability (Yes/No);
- perception of physical benefits (Yes/No);
- perception of cognitive benefits (Yes/No);
- perception of emotional benefits (Yes/No);
- satisfaction for the variety of exercises (Yes/No).

Both the experimental and the control group received a comprehensive neuropsychological and physical evaluation after 12 months.

The study was conducted according to the Declaration of Helsinki principles and was approved by the Local Ethics Committee. All data were collected from the IRCCS Don Carlo Gnocchi Foundation.

### Participants

Participants were recruited from the Memory Clinic of University Hospital of Careggi, (Florence, Italy) and from the Don Carlo Gnocchi Foundation (Florence, Italy). Participants were included in the study if they fulfilled core criteria for MCI (1) or VCI (3), based on clinical, neuroimaging, and neuropsychological information. Additional inclusion criteria were: (1) Mini Mental State Examination (MMSE) score >24; (2) age between 65 and 80 years old; (3) school attendance >3 years; (4) right-handed according to the Edinburgh Scale (40); (5) Italian language as mother tongue; (6) normal or corrected visual and auditory acuity; (7) preserved physical mobility or manual dexterity. According to the National Institute of Neurological and Communicative Disorders and Stroke and the Alzheimer's Disease and Related Disorders Association (NINCDS-ADRDA) and the National Institute of Neurological Disorders and Stroke and the Association Internationale pour la Recherche et l'Enseignement en Neurosciences (NINDS-AIREN) criteria (41, 42), patients with mild dementia were excluded. Other exclusion criteria were: intellectual deficiency, alcoholism or toxicomania, use of psychotropic medication known to impair cognition, presence or history of severe psychiatric disorders, presence or history of stroke, presence or history of a neurological disorder, and general anesthesia in the last 6 months. During the initial contact, the details of the study were explained and only individuals who agreed to participate for the duration of the study were retained. All participants signed a consent form prior to participation in the study.

### Tele-R Program: GOAL-App

The GOAL-app is a newly designed web-application (23, 43) that includes an *ad-hoc* weekly program that combines cognitive, physical, and social activities. The detailed explanation has been published in Martini et al. (34).

The first web-app prototype was implemented through a series of design and feedback loops with MCI/VCI specialists, patients with MCI/VCI and their caregivers. Before the beginning of the 8-weeks program, participants were trained to use the provided tablet autonomously by a multidisciplinary team.

**Figure 1:**
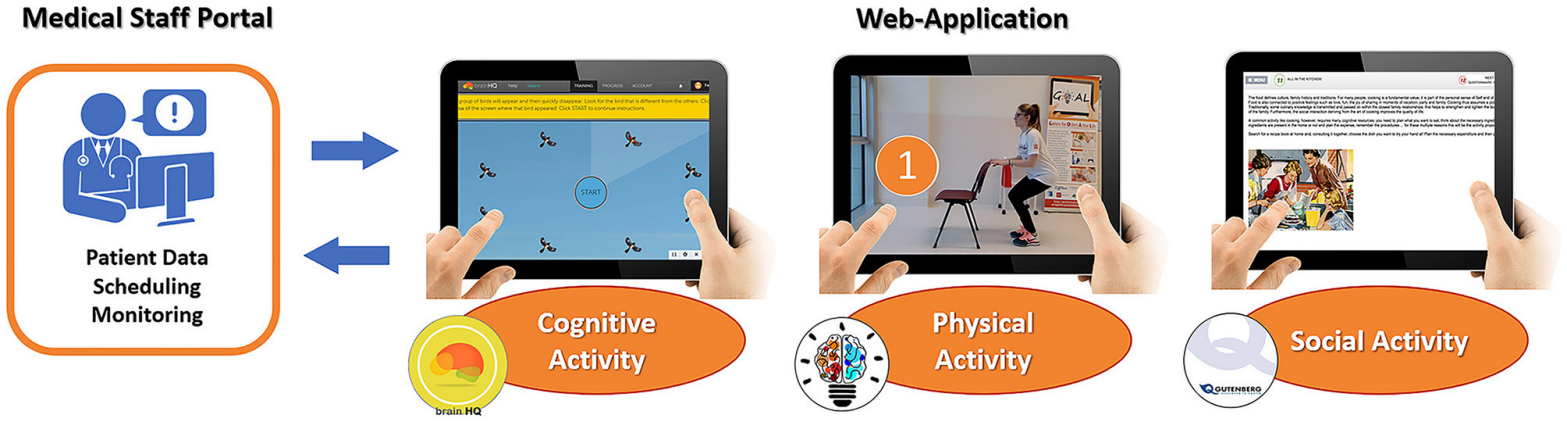
Functional architecture of GOAL-App.

The planning and the monitoring of each activity were accessible by the clinical team through the admin interface of the app. Each activity was implemented in three independent modules (Figure 1):

➢ The cognitive module integrated cognitive exercises from BrainHQ, a third-party platform developed by Posit Science [Posit Science Corporation, San Francisco, CA (44)]. Participants performed these exercises 3 days per week.
➢ The physical module included a training program of APA exercises (45), delivered through a guided video, to be performed 2 days per week.
➢ The caregiver module included suggestions on social activities to be carried out with the caregiver during the weekend. Patients were suggested to carry out one activity per week.

### Statistical Analysis

Feasibility of the Tele-R system was evaluated by means of distribution of patients' attrition from the screening phase to follow-up assessments (excluded and drop-out rates). In order to control for potential systematic bias due to the technological device, comparisons between patients that completed the study and drop-outs will be carried out for baseline characteristics (demographics, global cognitive and functional status, neuropsychological test scores, mood, and quality of life) by means of univariate analyses (Mann-Whitney *U*-tests and chi square tests).

Adherence to the Tele-R system was evaluated comparing drop-out distributions according to treatment groups (chi square test), and showing percentages and means of a rehabilitation adherence score (AS) of carried out activities (cognitive, social, and physical) within the treated group.

Patients' appreciation of the Tele-R system was analyzed through descriptive statistics of the *ad hoc* satisfaction questionnaire items.

## Results

**Figure 2:**
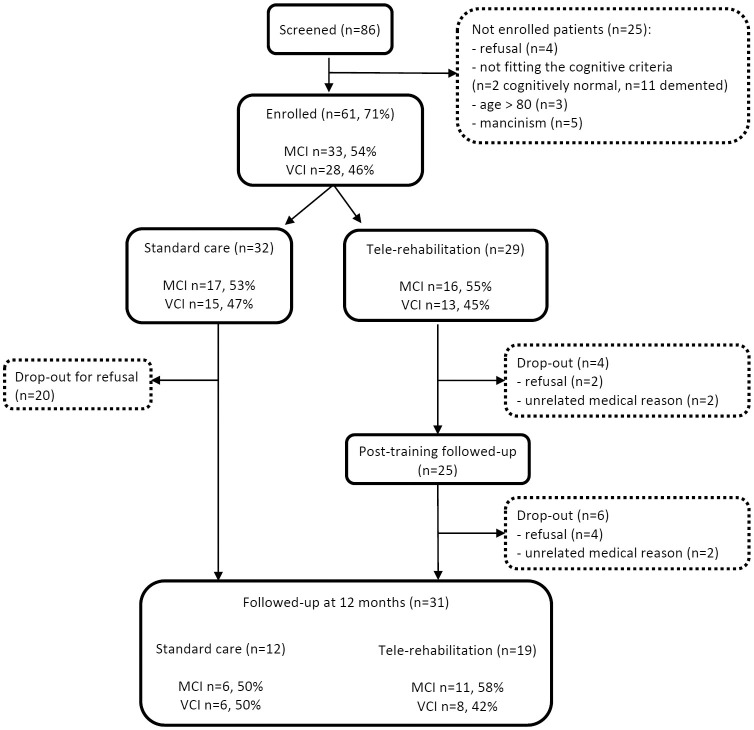
Flow diagram representing patients' attrition from the screening phase to follow-up assessments.

Flow diagram representing patients' attrition from the screening phase to follow-up assessments is reported in Figure 2. Eighty-six persons were contacted to enter the study, and 25 (29%) were not enrolled due to the following reasons: refusals (*n* = 4), not fitting the cognitive criteria (cognitively normal *n* = 2, demented *n* = 11), age >80 years (*n* = 3), and mancinism (*n* = 5). Of the remaining 61 patients, 32 (52%) were randomly assigned to the standard care group and 29 (48%) to the tele-R group. Among the standard care group, 20 (62.5%) patients dropped-out and refused to undergo the 12-month follow-up visit. In the tele-R group, four patients dropped-out during the training period (refusals *n* = 2, unrelated medical reasons *n* = 2), and further six patients completed the treatment and were evaluated at the post-training follow-up visit but declined the 12-month final visit (refusals *n* = 4, unrelated medical reasons *n* = 2).

**Figure 3:**
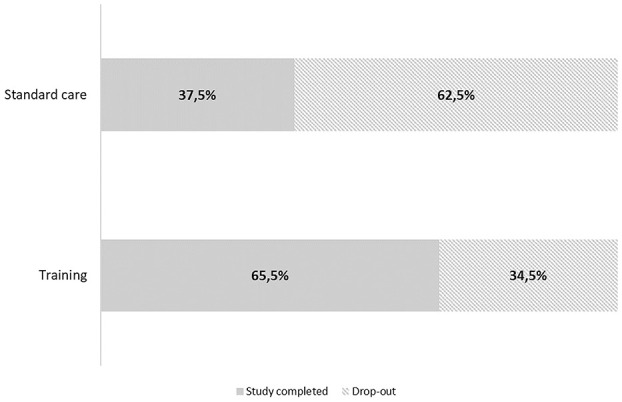
Drop-out distributions according to treatment groups (χ^2^ = 4.778, *p* = 0.029).

As shown in Figure 3, there was a statistically significant difference in drop-out rates between treated and non-treated patients (34.5 vs. 62.5%, respectively, χ^2^ = 4.778, *p* = 0.029). Thirty-one patients (51% of baseline enrolled cohort) completed the final 12-month visit (standard care group *n* = 12, the tele-R group *n* = 19).

Despite the high rate of drop-outs among enrolled patients, as shown in Table 1 comparisons between patients that completed the study and drop-outs showed not statistically significant differences in baseline demographics characteristics (age 74.2 ± 4.1 vs. 73.6 ± 3.9; years of education 10.3 ± 4.6 vs. 9.8 ± 4.6; global cognitive and functional status (MoCA score 20.9 ± 3.3 vs. 22.6 ± 3.7; ADCS/ADL score 75.2 ± 4.4 vs. 75.2 ± 3.6, neuropsychological data (see Table 1 for details), mood and quality of life (CESD score 14.9 ± 6.6 vs. 16.6 ± 6.9; physical component summary of SF-36 49.8 ± 9.4 vs. 46.4 ± 8.5, mental component summary of SF-36 45.9 ± 9.7 vs. 43.8 ± 8.3) except for sex. Females resulted in a statistically significant higher rate of drop-outs (66%) compared to males (27%, *p* = 0.003) (Table 1). Among the 23 females that dropped out, 17 (74%) were in the standard care group, while 6 (26%) were in the tele-rehabilitation one. Comparisons between males and females showed no statistically significant differences in age (74.6 ± 3.4 vs. 73.4 ± 4.3 years, respectively, *p* = 0.229), years of education (10.6 ± 4.8 vs. 9.7±4.4 years, respectively, *p* = 0.437), baseline MoCA demographically adjusted total score (20.9 ± 3.6 vs. 22.6 ± 3.6, respectively, *p* = 0.069), and baseline depressive symptoms as measured by the CESD scale total score (13.9 ± 5.2 vs. 17 ± 7.5, respectively, *p* = 0.076).

**Table 1:** Comparisons between patients that completed the study and drop-outs for the baseline characteristics.

			**Study completed**	**Drop-Out**	
		**Score range**	***N*** **=** **31**	***N*** **=** **30**	***p***
Age, years^*^	mean ± SD	-	74.2 ± 4.1	73,6 ± 3.9	0.563
Years of education^*^	mean ± SD	-	10.3 ± 4.6	9.8 ± 4.6	0.650
Sex^#^					
Females	*N*(%)	-	**12 (34%)**	**23 (66%)**	**0.003**
Males			**19 (73%)**	**7 (27%)**	
Cognitive impairment subtype (MCI)^#^	*N*(%)	-	55%	53%	0.906
Montreal Cognitive Assessment^*^	mean ± SD	0–30	20.9 ± 3.3	22.6 ± 3.7	0.070
FCSRT Immediate Free Recall^*^	mean ± SD	0–36	23.7 ± 6.3	24.4 ± 8.2	0.423
FCSRT Delayed Free Recall^*^	mean ± SD	0–12	8 ± 3.1	8.2 ± 3.9	0.248
Digit span forward^*^	mean ± SD	3–9	5.8 ± 1.2	5.7 ± 0.9	0.530
Digit span backward^*^	mean ± SD	3–9	4.3 ± 1.1	4.1 ± 1.1	0.464
Corsi span forward^*^	mean ± SD	3–9	5.3 ± 1.1	4.9 ± 0.8	0.180
Corsi span backward^*^	mean ± SD	3–9	4.6 ± 1	4.7 ± 1.1	0.668
Rey Complex Figure Test copy^*^	mean ± SD	0–36	30.9 ± 8.3	31.8 ± 5.9	0.893
Rey Complex Figure Test delayed recall^*^	mean ± SD	0–36	14.8 ± 7.5	20.1 ± 18.9	0.375
Modified card sorting Test (*errors*)^*^	mean ± SD	-	5.8 ± 4.8	7.1 ± 5.7	0.422
Trail Making Test, Part A (*time, seconds*)^*^	mean ± SD	-	39.1 ± 23.7	44.1 ± 34.1	0.808
Trail Making Test, Part B (*time, seconds*)^*^	mean ± SD	-	72.7 ± 74.6	58.2 ± 67.1	0.791
Stroop test (*time, seconds*)^*^	mean ± SD	-	18.1 ± 13.2	25.9 ± 16.6	0.054
Stroop test (*errors*)^*^	mean ± SD	-	3.4 ± 7	1.9 ± 5.8	0.479
Phonemic verbal fluency^*^	mean ± SD	-	31.4 ± 11.8	34.3 ± 10.3	0.419
Semantic verbal fluency^*^	mean ± SD	-	37.5 ± 8.9	35.5 ± 11.6	0.466
Activities of Daily Living Inventory^*^	mean ± SD	0–78	75.2 ± 4.4	75.2 ± 3.6	0.750
Center for Epidemiological Studies Depression scale^*^	mean ± SD	0–60	14.9 ± 6.6	16.6 ± 6.9	0.351
Physical component summary (36-Item Short Form Survey)^*^	mean ± SD	0–100	49.8 ± 9.4	46.4 ± 8.5	0.056
Mental component summary (36-Item Short Form Survey)^*^	mean ± SD	0–100	45.9 ± 9.7	43.8 ± 8.3	0.344

*FCSRT: Free and Cued Selective Reminding Test*.

* *Mann-Whitney U-tests*,

# *Chi square tests. Bold values represent a statistically significant difference in the distribution of the variable sex between the groups*.

Regarding the “cognitive impairment subtype” variable, it is worth noting that MCI and VCI were balanced between drop-outs and participants who completed the study, at different time point: at the enrollment, at the treatment allocation, and at the study termination (Table 1; Figure 2).

**Figure 4:**
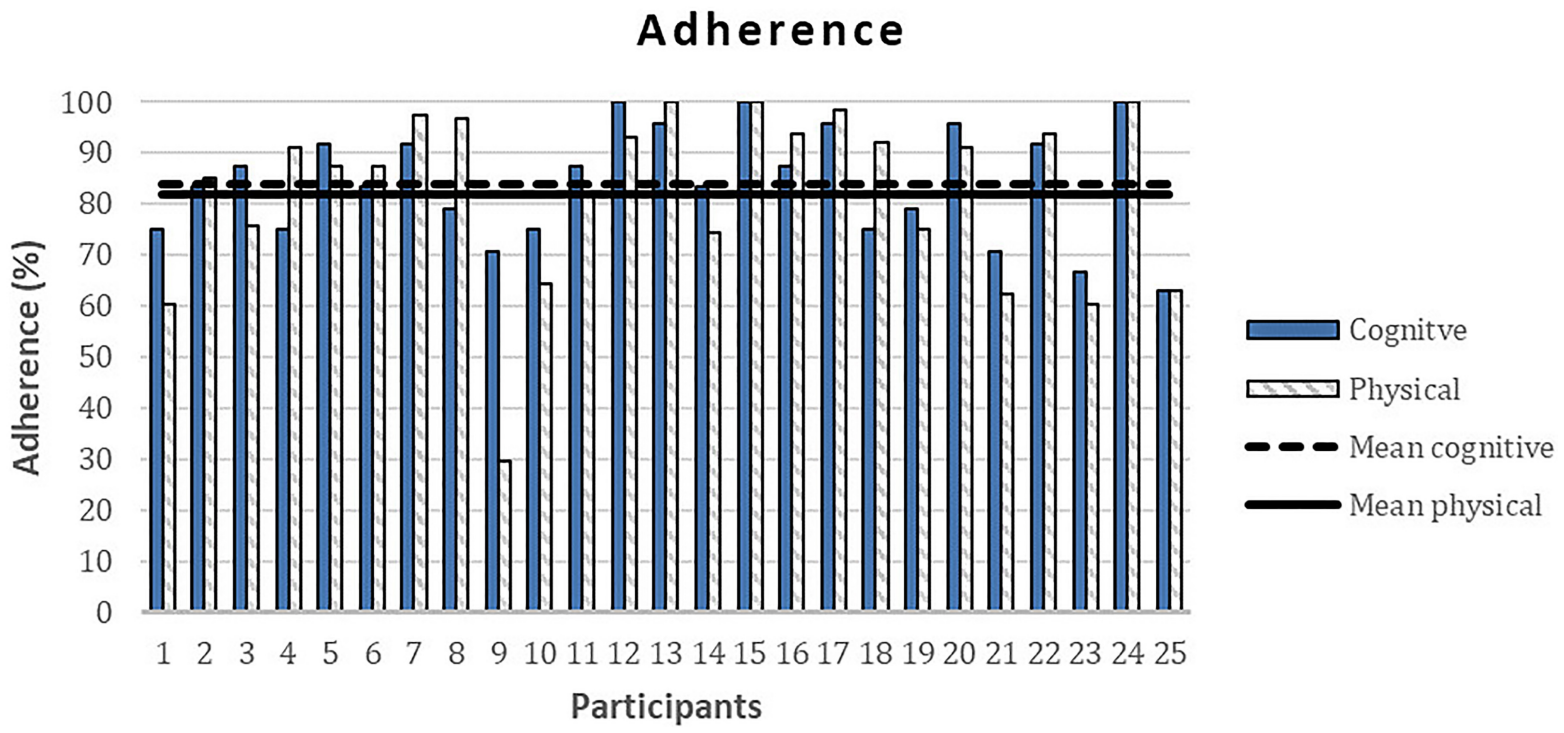
Adherence to the proposed activities in treated subjects.

The overall mean rehabilitation adherence score (SD) for the treated group was 84%. Particularly, participants showed a high adherence to the proposed activities with 85% for cognitive module and 83% for physical activity module (see Figure 4). Conversely, only one participant carried out the whole social module, the other participants did not complete any of the activities proposed in this module.

**Figure 5:**
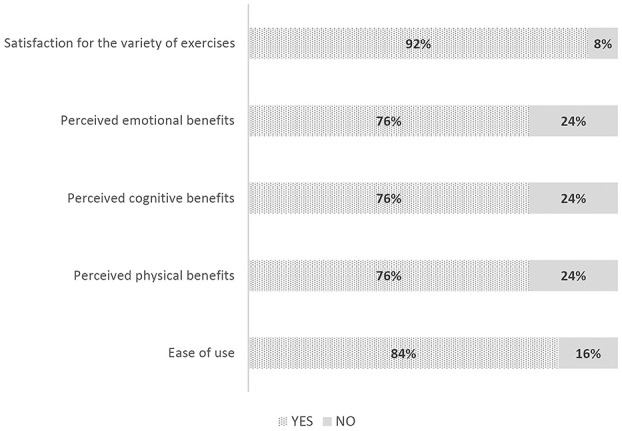
Results of the *ad hoc* satisfaction questionnaire.

Concerning results of the *ad hoc* satisfaction questionnaire, all participants judged the program as useful and their average level of appreciation of the treatment was good (range 1–5; mean = 3.7, SD = 0.8). As shown in Figure 5, 92% of participants reported to be satisfied with the variety of exercises, and 84% gave positive feedback in terms of ease of use. Particularly, 76% of patients reported a subjective perception of benefits regarding cognition, physical wellness, and emotional benefits after the 8-weeks program. Specifically, 60% of participants declared to prefer both cognitive and physical modules, 32% appreciated the cognitive module more, and the remaining 8% the physical module.

## Discussion

The proposed GOAL Tele-R system showed encouraging results in terms of feasibility, adherence, and appreciation in our cohort of MCI/VCI patients. Refusal rate was ~6% of the eligible patients, and drop-out rates resulted significantly higher in standard care than in treated patients, thus the proposed approach seemed not to discourage patients from participating.

Previous studies on the efficacy of a cognitive computer intervention program in patients with MCI reported no drop-out rates within the treated groups (46–48), thus corroborating the hypothesis of an overall good compliance of computer-based cognitive treatments. On the other hand, these training programs were quite short (~4 weeks), and drop-out rates were likely to increase in studies based on longer computer-assisted trainings. In a study by Belleville and colleagues, a drop-out rate of 15% was reported for an intervention administered in 8 weekly sessions of 120 min each (49).

Compared to these studies, our results showed a higher drop-out rate (34.5% in the tele-rehabilitation group and 62.5% in the standard care group), however, similar value of drop-out in the intervention group was reported by Makai (38%) (24) and De Cola (29%) (34). Causes of discontinuation have been listed in Figure 2 (refusal/unrelated medical reason). A possible explanation of drop-out in the tele-rehabilitation group may be due to a demanding approach both for the long duration and its multidimensional format. Indeed, a difficulty in introducing elderly people to a new technology, has been reported by other Authors (18, 20, 22, 24, 28, 34). Since normal or corrected visual and auditory acuity was requested at the inclusion, participants did not drop out for disperception. However, it is encouraging that the higher drop-out rate was found in the non-treated group, and that high overall adherence to the training program (84%) was documented for the treated group. It is possible that participants in the control group felt alone in counteracting their cognitive problems and in the absence of a treatment, they felt less motivated to continue the study. A possible solution may be represented by a combination of technical devices and personal interaction with health care professionals, in order to monitor and support patients' adherence to the program (34, 50).

No differences were found in baseline characteristics of drop-outs and participants who completed the study, except for sex. Since the two groups did not show differences in neuropsychological results, the absence of a selection bias based on cognitive status is confirmed. On the other hand, the higher rate of women who dropped out from the program compared to males is interesting and may reflect some gender-specific issue in the study compliance: it is possible that women encountered difficulties in balancing the treatment demands and the management of their role within the family, e.g., household activities. In this regard, no differences were found between males and females on baseline characteristics. Different results were obtained by De Cola and Colleagues in a study aimed at assessing usability and patients' satisfaction of a teleassistance program for frail elderly people. They found that a reduced adherence in the teleassistance program was associated with male gender, older age, urban residence, and with the application of the isolated telemonitoring program, when not associated with the telecounselling through an audio-video conference (34). Differently from the study protocol of De Cola, in our study, a control group was compared to the treated group. Nowadays, web-technologies provide high tailored open sources services and in the proposed work it is possible to observe a good level of appreciation and an overall positive feedback for usability. This is probably due to a deep customization of the web-application. Furthermore, our results showed that patients reported a subjective perception of cognitive, emotional, and physical benefits due to the training. In this regard, Cotelli and Colleagues argue that currently the available evidence is insufficient to draw conclusions about the effects of tele-rehabilitation on cognition, health related quality of life or participant satisfaction, highlighting that the quality of the clinical studies' designs need to be improved. An encouragement to the tele-rehabilitation use comes from a recent review that confirms Virtual-reality technology as a very effective tool for cognitive assessment and recovery in patients with cognitive impairment (51). Moreover, a recent meta-analysis conducted on Exergames (Physically-active video games) concluded that benefits were observed with this kind of treatment for both healthy elderly and clinical populations with cognitive impairments (52).

This study also had several potential limitations. First, patients underwent different types of exercises during the course of the 8 weeks, and some reported dissatisfaction for the variety of proposed activities. To overcome this limitation, an additional integration of other exercises could reduce the repetitiveness. Second, the low adherence to the social module during the weekend might be the result of an over demanding request, whose positive impact was probably underestimated by some of the patients. A possible solution may be to improve patients' engagement and motivational support through a strategy based on periodical “human” contact (e.g., telephone calls, text messages, or email) and feedback (53, 54). Another important limitation of this project is the low level of involvement of the caregiver during the patient's activities. Since caring for a person with cognitive decline may have negative consequences for caregivers, several studies highlight the importance of their support (55, 56), in line with the Chronic Care Model (57, 58). Unfortunately, the comprehensive involvement of the caregiver is often difficult and the use of care technology with caregivers is still limited in daily practice (59, 60). Since follow-up data are not yet analyzed, currently it is impossible to state whether our results on feasibility, adherence and satisfaction of the tele-rehabilitation program have been influenced or not by the progression of the disease. We evaluated adherence and feasibility with objective measures and satisfaction through an *ad hoc* satisfaction questionnaire. We are confident that these tools are suitable for this scope. However, in this context, a standardized protocol is not yet available.

Taking into account the relatively low costs and easy accessibility of this e-health intervention, the GOAL Tele-R system seems to be an efficient and promising program to take care of patients with MCI/VCI. However, further studies must quantitatively assess the efficacy of this system, in terms of counteracting cognitive decline.

To conclude, the GOAL Tele-R system seems suitable to provide a multidimensional rehabilitation program, and may represent an enabling technology in the healthcare sector that allows a customized person-centered intervention.

## Data Availability Statement

The raw data supporting the conclusions of this article will be made available by the authors, without undue reservation.

## Ethics Statement

The studies involving human participants were reviewed and approved by Careggi University-Hospital Local Ethics Committee. The patients/participants provided their written informed consent to participate in this study.

## Author Contributions

IEM, ES, LF, GLu, LB, FB, FV, and CM developed the original concept of the trial. LF, FB, GLu, FV, and CM drafted the original protocol. LF, IEM, FG, SP, GLu, FB, FV, and CM developed the design. IEM, ES, LF, GLu, FB, and CM developed the methodology. ES, IEM, SP, LF, FB, FV, and CM developed the analysis plan. IEM, ES, LF, GLo, FG, FB, FV, and CM adapted the trial proposal as a protocol paper. IEM, ES, LF, SP, FV, GLo, SS, and CM did manuscript writing. All authors reviewed and commented on drafts of the protocol and paper. All authors read and approved the final manuscript.

## Conflict of Interest

The authors declare that the research was conducted in the absence of any commercial or financial relationships that could be construed as a potential conflict of interest.
